# Poor sleep quality is associated with increased arterial stiffness in Japanese patients with type 2 diabetes mellitus

**DOI:** 10.1186/s12902-015-0026-1

**Published:** 2015-06-18

**Authors:** Yusuke Osonoi, Tomoya Mita, Takeshi Osonoi, Miyoko Saito, Atsuko Tamasawa, Shiho Nakayama, Yuki Someya, Hidenori Ishida, Akio Kanazawa, Masahiko Gosho, Yoshio Fujitani, Hirotaka Watada

**Affiliations:** Department of Metabolism and Endocrinology, Juntendo University Graduate School of Medicine, 2-1-1 Hongo, Bunkyoku, 113-8421 Tokyo Japan; Center for Molecular Diabetology, Juntendo University Graduate School of Medicine, 2-1-1 Hongo, Bunkyoku, 113-8421 Tokyo Japan; Center for Therapeutic Innovations in Diabetes, Juntendo University Graduate School of Medicine, 2-1-1 Hongo, Bunkyoku, 113-8421 Tokyo Japan; Sportology Center, Juntendo University Graduate School of Medicine, 2-1-1 Hongo, Bunkyoku, 113-8421 Tokyo Japan; Naka Memorial Clinic, 745-5, Nakadai, Naka City, 311-0113 Ibaraki Japan; Department of Clinical Trial and Clinical Epidemiology, Faculty of Medicine, University of Tsukuba, 1-1-1, Tennodai, Tsukuba, 305-8575 Ibaraki Japan

**Keywords:** Sleep quality, Arterial stiffness, Type 2 diabetes mellitus, Japanese

## Abstract

**Background:**

While poor sleep quality can worsen cardiovascular risk factors such as glucose and lipid profiles in patients with type 2 diabetes mellitus (T2DM), the relationship between sleep quality and atherosclerosis remains largely unknown. The aim of this study was to examine this relationship.

**Methods:**

The study participants comprised 724 Japanese T2DM outpatients free of history of cardiovascular diseases. The relationships between sleep quality (assessed by the Pittsburgh Sleep Quality Index (PSQI)) and various clinical and laboratory parameters were investigated.

**Results:**

The mean PSQI was 5.1 ± 3.0 (±SD). Patients were divided into three groups based on the total PSQI score; subjects with good sleep quality (*n* = 462), average sleep quality (*n* = 185), and poor sleep quality (*n* = 77). In the age/gender-adjusted model, patients with poor sleep quality tended to be obese, evening type and depressed. However, other lifestyles showed no significant trends. Alanine aminotransferase, fasting blood glucose, HbA1c, systolic blood pressure, urinary albumin excretion, and brachial-ankle pulse wave velocity (baPWV) tended to be higher in patients with poor sleep quality. High baPWV was the only parameter that correlated with poor sleep in a model adjusted for several other lifestyle factors.

**Conclusions:**

Our study indicates that poor sleep quality in T2DM patients correlates with increased arterial wall stiffness, a marker of atherosclerosis and a risk factor for cardiovascular diseases.

## Background

The onset and development of type 2 diabetes mellitus (T2DM) is associated with numerous lifestyle factors. Furthermore, the risk of cardiovascular disease (CVD) and microvascular events in patients with T2DM correlate with such lifestyle factors [1, 2]. Evidence suggests that poor sleep quality is an important pathological factor in the onset of T2DM [3–9] and also in CVD [10] in non-T2DM subjects. On the other hand, patients with T2DM are reported to have various sleep abnormalities compared to healthy control subjects [11]. Furthermore, recent studies reported that short sleep and/or lack of deep sleep are risk factors for hyperglycemia [12–14], hyperlipidemia [15, 16] and renal dysfunction [17]. However, little is known at present about the relation between poor sleep quality and progression of atherosclerosis in patients with T2DM.

The brachial-ankle pulse wave velocity (baPWV) is a noninvasive technique for assessment of arterial wall stiffness. It is often used clinically to evaluate the state of atherosclerosis and prediction of CVD in both non-diabetic [18, 19] and T2DM patients [20, 21].

The purpose of the present study was to determine the impact of poor sleep quality on various parameters, including arterial wall stiffness, in patients with T2DM.

## Methods

### Subjects

The subjects of this cohort study were recruited from the Diabetes Outpatient Clinic of Juntendo University (Tokyo, Japan), Naka kinen Clinic (Naka, Japan), and Secomedic Hospital (Funabashi, Japan) [22]. The inclusion criteria were as follows: *1)* T2DM male and female patients, *2)* ≥25 years of age and <70 years of age, and *3)* signing consent form for participation in the study. The following exclusion criteria were also applied: *1)* type 1 or secondary diabetes, *2)* presence of severe infection, recent surgery, and severe trauma, *3)* history of myocardial infarction, angina pectoris, or stroke, *4)* chronic renal failure requiring hemodialysis, *5)* liver cirrhosis, *6)* moderate or severe heart failure (NYHA/New York Heart Association stage III or higher), *7)* active malignancy, *8)* pregnancy, lactation, or possible pregnancy, *9)* patients judged as ineligible by the clinical investigators.

A total of 1032 consecutive subjects were screened. Among them, 906 patients who met the above eligibility criteria were invited to participate in the present study. After providing information on the purpose and procedures of the study, 736 patients with T2DM were enrolled in this study between June 2013 and January 2014. The study was approved by the Institutional Review Board of our hospital and conducted in accordance with the principles described in the Declaration of Helsinki. All patients provided written informed consent prior to participation. The study was registered on the University Hospital Medical Information Network Clinical Trials Registry (UMIN000010932).

### Questionnaire survey

The self-administered questionnaire survey used in the present study was the Pittsburgh Sleep Quality Index (PSQI) [22, 23], which has been validated previously [22]. It is used to evaluate sleep quality and consists of 18 items that in turn are comprised of 7 components covering subjective sleep quality, sleep duration, sleep onset, habitual sleep efficiency, sleep disturbances, use of sleeping medications, and daytime dysfunction. Each component is weighted equally on a 0–3 scale, and summed to yield the total PSQI score (range, 0–21), with the highest score denoting worst sleep quality. Based on the total PSQI score, patients were divided into three groups; the “Good sleep quality group” with PSQI score of ≤5: “Average sleep quality group” with PSQI 6–8, and “Poor sleep quality group” with PSQI ≥9 [14].

We also used the Morning Evening Questionnaire (MEQ) [24] to evaluate morningness and eveningness in individuals. A high MEQ score represents morning type. The participating patients also completed the BDI (Beck Depression inventory)-II, which is a 21-item questionnaire that assesses hopelessness, irritability, cognition, guilt, fatigue, weight loss, and sexual interest, representing depression-related symptoms [25]. A high BDI score represents depressive state. Dietary habits during the preceding month were also assessed by the self-administered Diet History Questionnaire (BDHQ). Briefly, the 4-page structured BDHQ includes questions on selected foods and is designed to estimate the dietary consumption of 56 food and beverage items [26]. Physical activity level was assessed with the 4-question International Physical Activity Questionnaire (IPAQ) [27], and the results were expressed as metabolic equivalent scores (METs-hour-week^−1^). In the above questionnaires, workers were defined as full-time employees or shift workers [22]. The subjects were also divided into non-smokers, former smokers or current smokers [22].

### Blood and urine tests

Blood samples were collected at the Outpatient Clinic after overnight fast. Liver and renal function tests, lipid profile, HbA1c (National Glycohemoglobin Standardization Program), and blood glucose were measured with standard techniques. UAE was measured by the latex agglutination assay using a spot urine sample. The estimated glomerular filtration rate (eGFR) was calculated by the formula: eGFR (ml/min per 1.73 m^2^) =194× Age^-0.287^× serum creatinine^-0.1094^ (×0.739 for females) [28].

### Measurement of baPWV

baPWV was measured using an automatic waveform analyzer (BP-203RPE; Colin Medical Technology, Komaki, Japan), as described previously [22, 29]. Briefly, recording was performed with the patients in the supine position after 5-min rest. Occlusion and monitoring cuffs were placed snugly around both areas in the upper and lower extremities. The pressure waveforms were then recorded simultaneously from the brachial arteries by the oscillometric method. All scans were automatically conducted by well-trained investigators who were blinded to the clinical information. Previous studies confirmed the validity and reproducibility of baPWV measurements [30]. The ankle-brachial index was measured in all participants. A resting ankle-brachial index ≤ 0.90 was considered to reflect the presence of peripheral artery disease. Subjects with baPWV-determined peripheral artery disease underwent computed tomographic angiography, magnetic resonance angiography or catheter angiography to confirm the diagnosis of peripheral artery disease. Arterial stenotic lesions represented arterial lumen narrowing of ≥50 %. Six patients with peripheral artery disease were excluded from analysis.

### Statistical analysis

Results are presented as mean ± SD or median (interquartile range: 25 to 75 %) for continuous variables or number (proportion) of patients for categorical variables. Several parameters were logarithmically transformed to approximate normal distribution. Trend association across the three PSQI score-based groups was evaluated by linear regression analysis for continuous variables and logistic regression analysis for categorical variables. We developed three models to evaluate the trend. The first model was unadjusted, the second was adjusted for age and gender but not eGFR, and the third model was adjusted for age, gender, body mass index (BMI), MEQ, BDI-II, energy intake, smoking pattern, alcohol consumption and IPAQ. The final model for baPWV was adjusted for age, gender, BMI, MEQ, BDI-II, energy intake, smoking pattern, alcohol consumption, IPAQ, systolic blood pressure (SBP), HbA1c, total cholesterol, high-density lipoprotein cholesterol (HDL) cholesterol, triglyceride and sleep duration. Statistical tests were two-sided with 5 % significant level. All analyses were performed using the SAS software version 9.3 (SAS Institute, Cary, NC).

## Results

Among the 736 participating patients, 12 did not complete the PSQI questionnaires and they were thus excluded from this analysis. The characteristics of the remaining 724 Japanese patients with T2DM are shown in Table 1. The mean age was 57.8 ± 8.6 years, and 62.9 % subjects were males. The mean HbA1c was 7.0 ± 1.0 %, and the estimated duration of T2DM was 9.9 ± 7.2 years. Most subjects had previously attended educational programs about diet and exercise therapy and received appropriate medical treatments. Thus, blood glucose levels, lipid profile, and SBP were well controlled.

**Table 1:** Patients’ characteristics (n = 724)

Age (years)	57.8 ± 8.6
Gender (male)	456 (62.9)
Estimated history of diabetes (years)	9.9 ± 7.2
Body mass index (kg/m^2^)	24.6 ± 4.1
HbA1c (%)	7.0 ± 1.0
HbA1c (mmol/mol)	52.5 ± 10.8
Fasting blood glucose (mg/dl)	134 ± 31
Systolic blood pressure (mmHg)	127 ± 14
Diastolic blood pressure (mmHg)	77 ± 11
Total cholesterol (mg/dL)	185 ± 28
HDL-cholesterol (mg/dL)	59 ± 14
Triglyceride (mg/dL)	100 [70,152]
AST (U/L)	21 [18, 27]
ALT (U/L)	22 [16, 33]
γ-GTP (U/L)	25 [17,39]
Uric Acid (mg/dl)	5.5 ± 1.2
eGFR (ml/min/1.73 m^2^)	78 ± 18
UAE (mg/g creatinine)	10 [6, 23]
baPWV (cm/s)	1543 ± 279
Morningness-Eveningness Questionnaire	57.4 ± 7.3
Pittsburgh Sleep Quality Index	5.1 ± 3.0
Beck Depression inventory -II	9.9 ± 7.6
Energy intake (kcal/day)	1713 ± 582
Physical activity (Mets/h/week)	42.8 ± 70.5
Sleep duration (hours)	6.4 ± 1.2
Current smoker (yes)	174 (24.0)
Alcohol (g/day)	12.3 ± 21.5
On treatment for (n/%)	
Diabetes	620 (85.5)
Hypertension	346 (47.7)
Dyslipidemia	442 (61.0)

Data are mean ± SD or number (proportion) of patients

*ALT* alanine aminotransferase, *AST* aspartate aminotransferase, *baPWV* brachial-ankle pulse wave velocity, *eGFR* estimated glomerular filtration rate, *HDL-C* high-density lipoprotein-cholesterol, *UAE* urinary albumin excretion, *γ-GTP* γ-glutamyl transpeptidase

Based on the results of the PSQI, 462 individuals were categorized into the “Good sleep quality group”, 185 into the “Average sleep quality group”, and 77 into the “Poor sleep quality group” (Table 2). Subjects with poor sleep quality tended to be younger, female and obese, and had low MEQ and high BDI-II in the unadjusted model. In the age/gender-adjusted model, they still tended to be more obese, had low MEQ and high BDI-II. On the other hand, no significant trends were found in other lifestyles including energy intake, smoking, alcohol consumption and IPAQ. In both the unadjusted and age/gender-adjusted models, subjects with poor sleep quality type were likely to go to bed late at night, wake up late, sleep for short time and less frequently had breakfast. However, these findings were not observed in the model adjusted for age, gender, BMI, MEQ, BDI-II, energy intake, smoking, alcohol consumption and IPAQ.

**Table 2:** Characteristics of subjects of the three sleep quality groups

Variable	Sleep quality group			Unadjusted	Model 1	Model 2
	Good (*n* = 462)	Average (*n* = 185)	Poor (*n* = 77)			
PSQI	3.4 ± 1.3	6.8 ± 0.8	11.5 ± 2.9	-	-	-
Sleep duration (hours)	6.8 ± 1.0	6.0 ± 1.0	5.3 ± 1.1	−13.60^***^	−13.12^***^	−13.72^***^
Wake time, A.M.	6:00 [5:30,6:30]	6:00 [5:30,6:30]	6:00 [5:30,7:00]	2.20^*^	2.06^*^	0.79
Bed time, P.M.	23:00 [22:00,23:30]	23:30 [22:30,24:00]	23:30 [22:00,24:00]	2.91^**^	2.12^*^	0.79
MEQ	58.6 ± 7.1	55.4 ± 7.2	54.9 ± 7.2	−5.81^***^	−5.14^***^	-
BDI	8.0 ± 6.7	12.5 ± 7.8	15.1 ± 8.4	9.65^***^	9.23^***^	-
Energy intake (kcal/day)	1692 ± 542	1760 ± 646	1726 ± 650	1.00	1.70	-
Current smoking (yes)	114 (24.7)	42 (22.7)	17(22.1)	−0.64	−0.44	-
Alcohol	12.5 ± 21.1	10.2 ± 20.9	16.3 ± 24.7	0.59	1.76	-
Physical activity (kcal/day)	41.6 ± 65.1	47.9 ± 86.2	38.7 ± 59.2	0.22	0.42	-
Age (years)	58.4 ± 8.6	56.3 ± 8.5	57.2 ± 8.0	−2.25^*^	-	-
Gender (male)	304 (65.8)	111 (60.0)	40(51.9)	−2.48^*^	-	-
Body mass index (kg/m^2^)	24.4 ± 4.0	24.8 ± 4.1	25.8 ± 4.4	2.93^**^	2.21^*^	-
Estimated duration of diabetes (years)	10.0 ± 7.4	9.2 ± 6.1	10.8 ± 8.6	0.16	0.75	−0.25
Diabetes medication (yes)	395 (85.5)	156 (84.3)	68 (88.3)	0.34	0.41	0.37
Hypertension medication (yes)	212 (45.9)	93 (50.3)	41 (53.2)	1.42	1.70	0.61
Dyslipidemia medication (yes)	290 (62.8)	103 (55.7)	48 (62.3)	−0.84	−0.94	−1.30
Anti-platelet (yes)	14 (3.0)	4 (2.2)	5 (6.5)	1.00	1.46	1.18
Working (yes)	334 (72.3)	136 (73.5)	62 (80.5)	1.35	1.50	1.11
Shift worker (yes)	43 (9.3)	23 (12.4)	12 (15.6)	1.84	1.41	1.60
Breakfast time, A.M.	7:00 [6:30,7:30]	7:00 [6:30,7:30]	7:18 [6:30,8:00]	3.75^***^	3.30^**^	1.96
Dinner time, P.M.	19:00 [18:30,19:30]	19:00 [18:30,20:00]	19:00 [18:30,20:00]	3.32^***^	3.00^**^	1.90
Time of breakfast (/week)	6.7 ± 1.0	6.4 ± 1.5	6.3 ± 1.7	−3.36^***^	−3.16^**^	−0.64
Late evening snack (yes)	178 (38.5)	76 (41.1)	34 (44.2)	1.02	0.87	0.14

Data are mean ± SD or median (range: 25 to 75 %) or number of subjects

Unadjusted and Adjusted *Ptrend* values for linear trends across three groups are based on linear regression analysis for continuous variables or logistic regression analysis for categorical variables. Model 1 and Adjusted values for linear trends across three groups are based on linear regression analysis for continuous variables or logistic regression analysis for categorical variables adjusted for age and gender. Model 2 and Adjusted *Ptrend* values for linear trends across three groups are based on linear regression analysis for continuous variables or logistic regression analysis for categorical variables adjusted for age, gender, BMI, morningness-eveningness questionnaire, Beck Depression inventory, energy intake, alcohol intake, current smoking, and physical activity

^*^ *P* < 0.05; ^**^ *P* < 0.01; ^***^ *P* < 0.001

With regard to clinical and laboratory data, patients with poor sleep quality tended to have high levels of alanine aminotransferase (ALT), total cholesterol, fasting blood glucose, HbA1c, SBP and UAE in the unadjusted model (Table 3). Even in the age/gender adjusted model, ALT, fasting blood glucose, HbA1c, SBP, UAE, and baPWV tended to be higher in patients with poor sleep quality than the other two groups. Intriguingly, a high baPWV level was the only factor related to patients with poor sleep quality in the adjusted model by age, gender, BMI, MEQ, BDI-II, energy intake, smoking, alcohol consumption and IPAQ. Furthermore, baPWV tended to be higher in patients with poor sleep quality than the other two groups even in SBP, HbA1c, total cholesterol, HDL- cholesterol, triglyceride and sleep duration, which are potential risk factors for arterial stiffness, and age, gender, BMI, MEQ, BDI-II, energy intake, smoking consumption, alcohol consumption and IPAQ adjusted model.

**Table 3:** Clinical characteristics of the subjects of the three sleep quality groups from the results of regression model

Variable	Sleep quality group			Unadjusted	Model 1	Model 2	Model 3
	Good (*n* = 462)	Average (*n* = 185)	Poor (*n* = 77)				
AST (U/L)	21 [18, 27]	21 [18, 26]	22 [18, 27]	1.15	1.30	0.57	-
ALT (U/L)	22 [16,33]	22 [17,33]	25 [17,35]	2.18^*^	2.00^*^	1.07	-
γ-GTP (U/L)	25 [17,37]	25 [18,42]	26 [16,42]	0.95	1.40	0.03	-
Uric acid (mg/dl)	5.5 ± 1.2	5.4 ± 1.2	5.4 ± 1.4	−0.86	−0.21	−1.13	-
eGFR (ml/min/1.73 m^2^)	78 ± 18	79 ± 18	78 ± 19	0.65	-	0.55	-
Total cholesterol (mg/dl)	184 ± 28	188 ± 28	189 ± 25	2.04^*^	1.66	1.63	-
HDL-C (mg/dl)	59 ± 14	59 ± 14	61 ± 14	0.60	0.47	0.77	-
Triglycerides (mg/dl)	99 [68,150]	102 [72,148]	105 [74,158]	1.23	1.09	0.10	-
Fasting blood glucose (mg/dl)	132 ± 31	136 ± 31	141 ± 32	2.61^**^	2.41^*^	1.21	-
HbA1c (%)	6.9 ± 1.0	7.1 ± 1.1	7.1 ± 0.8	2.56^*^	2.01^*^	1.38	-
HbA1c (mmol/mol)	51.7 ± 10.6	54.0 ± 11.9	54.1 ± 9.0	2.56^*^	2.01^*^	1.38	-
Systolic BP (mmHg)	126 ± 14	127 ± 15	131 ± 13	3.07^**^	3.06^*^	1.95	-
Diastolic BP (mmHg)	77 ± 11	77 ± 11	79 ± 12	1.53	1.53	0.61	-
UAE (mg/g creatinine)	10 [6,21]	10 [6,25]	16 [7,40]	2.19^*^	2.23^*^	1.25	-
baPWV (cm/s)	1523 [1500, 1547]	1570 [1533, 1606]	1604 [1547, 1661]	-	3.01^**^	2.79^**^	2.58^*^

Data are mean ± SD or median (range: 25 to 75 %). baPWV values are age- and gender-adjusted values (95 % confidence interval)

Unadjusted and Adjusted *Ptrend* values for linear trends across three groups are based on linear regression analysis for continuous variables or logistic regression analysis for categorical variables. Model 1 and Adjusted *Ptrend* values for linear trends across three groups are based on linear regression analysis for continuous variables or logistic regression analysis for categorical variables adjusted for age and gender. Model 2 and Adjusted *Ptrend* values for linear trends across three groups are based on linear regression analysis for continuous variables or logistic regression analysis for categorical variables adjusted for age, gender, BMI, morningness-eveningness questionnaire, Beck Depression inventory, energy intake, alcohol intake, current smoking, and physical activity. Model 3 and Adjusted *Ptrend* values for linear trends across three groups are based on linear regression analysis for continuous variables adjusted for age, gender, BMI, morningness-eveningness questionnaire, Beck Depression inventory, energy intake, alcohol intake, current smoking, physical activity, systolic BP, HbA1c, total cholesterol, HDL-cholesterol, triglyceride and sleep duration

*ALT* alanine aminotransferase, *AST* aspartate aminotransferase, *baPWV* brachial-ankle pulse wave velocity, *BP* blood pressure, *eGFR* estimated glomerular filtration rate, *UAE* urinary albumin excretion, *HDL-C* high-density lipoprotein-cholesterol, *ɤ-GTP* ɤ-glutamyl transpeptidase

^*^ *P* < 0.05; ^**^ *P* < 0.01; ^***^ *P* < 0.001

## Discussion

The main finding of this study was that poor sleep quality correlated with arterial stiffness in patients with T2DM, even after adjustment for other lifestyle factors and risk factors for atherosclerosis. Another important finding was that poor sleep quality also correlated with various cardiovascular risk factors, such as poor glycemic control, high lipid profile, high SBP, and high ALT and UAE. However, these relationships disappeared after adjustment for other lifestyle factors.

Our study showed that poor sleep quality was associated with increased arterial stiffness after adjustment for multi-covariates including other life style factors. This association was still significant even after adjustment for potential risk factors for arterial stiffness in addition to other life style factors. These findings suggest that poor sleep quality may have some direct influence on arterial stiffness independent of cardiovascular risk factors. While the potential mechanism(s) linking poor sleep quality and arterial stiffness remains largely unknown, high levels of catecholamines due to poor quality and/or duration of sleep may play a role in the progression of arterial stiffness, as reported previously [31, 32]. In this regard, one previous study demonstrated that sympathetic activation may negatively influence arterial stiffness in human, independent of BP [33]. High catecholamine levels induced by sympathetic nerve activation may promote smooth muscle cell proliferation and fibrosis, which in turn contribute to structural changes in the arterial wall.

Our study also showed that a large proportion of T2DM had a PSQI score of more than 6 points, confirming the findings of previous studies [13, 14, 16]. In addition, patients with poor sleep quality, tended to have high levels of ALT, total cholesterol, fasting blood glucose, HbA1c, SBP and UAE, which is almost similar to the findings of previous studies [13, 14, 16]. However, in the above previous studies, other life style factors, such as diet, physical activity, chronotype and depressive state, were not fully taken into consideration. In the present study, which checked other life style factors, we investigated the relationship between poor sleep quality and cardiovascular risk factors. The results showed no relation between poor sleep quality and any of the cardiovascular risk factors. This finding suggests that various life style factors, in addition to poor sleep quality, contribute to worsening cardiovascular risk factors. In fact, patients with poor sleep quality tended to be more evening type and in depressive state. Recently, we and other groups reported the relationship of evening type to inadequate glycemic control in patients with T2DM [22, 34, 35]. In addition, depressive status could also negatively affect glucose metabolism based on the findings that increased counter-regulatory hormones present in the depressive state negatively affect glucose metabolism [36]. Thus, these factors in patients with poor sleep can negatively affect glucose metabolism.

The present study has certain limitations. First, the cross-sectional design does not allow inference of causal relationship between sleep quality and arterial stiffness.

Second, we evaluated lifestyles including PSQI by self-reported questionnaires. The results could be influenced by social desirability and recall bias. Third, the validity and reproducibility of the lifestyle patterns identified in this study were not confirmed, although we used valid and reliable life-related self-reported questionnaires. Fourth, we only used evaluation of arterial stiffness to assess atherosclerosis. It is important to validate the current findings using other techniques. The exclusion of subjects with history of CVD may have influenced the results. However, we are currently conducting long-term follow-up study that focuses on lifestyle and onset of primary CVD in the same cohort. This study might provide further information about the relationship between sleep quality and primary CVD in T2DM patients. Finally, we cannot exclude the possible effects of other lifestyle patterns that were not assessed in this study.

## Conclusions

In conclusion, our study demonstrated that poor sleep quality in T2DM patients correlates with increased arterial stiffness, a marker of atherosclerosis and a risk factor for CVD. While long-term prospective studies are needed to confirm the study findings, the findings suggest that poor sleep quality is an important target of intervention in order to achieve appropriate management of cardiovascular risk factors and prevent the progression of atherosclerosis in patients with T2DM.

## Abbreviations

ALT: Alanine aminotransferase
BaPWV: Brachial-ankle pulse wave velocity
BDI: Beck depression inventory
BDHQ: Brief, self-administered diet history questionnaire
BMI: Body mass index
BP: Blood pressure
CVD: Cardiovascular disease
eGFR: Estimated glomerular filtration rate
γ-GTP: γ-glutamyl transpeptidase
HDL: High-density lipoprotein-cholesterol
IPAQ: International Physical Activity Questionnaire
MEQ: Morningness-eveningness questionnaire
NGSP: National Glycohemoglobin Standardization Program
PSQI: Pittsburgh Sleep Quality Index
T2DM: Type 2 diabetes mellitus
UAE: Urinary albumin excretion
